# Nutritional interventions in treating menopause-related sleep disturbances: a systematic review

**DOI:** 10.1093/nutrit/nuad113

**Published:** 2023-09-11

**Authors:** Dominik Polasek, Nayantara Santhi, Pamela Alfonso-Miller, Ian H Walshe, Crystal F Haskell-Ramsay, Greg J Elder

**Affiliations:** Northumbria Sleep Research, Northumbria University, Newcastle upon Tyne, UK; Northumbria Sleep Research, Northumbria University, Newcastle upon Tyne, UK; Northumbria Sleep Research, Northumbria University, Newcastle upon Tyne, UK; Department of Sport, Exercise and Rehabilitation, Northumbria University, Newcastle, UK; Department of Psychology, Faculty of Health and Life Sciences, Northumbria University, Newcastle upon Tyne, UK; Northumbria Sleep Research, Northumbria University, Newcastle upon Tyne, UK

**Keywords:** menopause, nutrition, nutritional intervention, sleep

## Abstract

**Context:**

Sleep disturbances are a core symptom of menopause, which refers to the permanent cessation of menstrual periods. Nutritional interventions may alleviate menopause-related sleep disturbances, as studies have shown that certain interventions (eg, tart cherry juice, or tryptophan-rich foods) can improve relevant aspects of sleep.

**Objective:**

The aim of this systematic review was to examine the effect of nutritional interventions for menopause-related sleep disturbances, in order to inform the subsequent development of specific interventional trials and assess their potential as a treatment for menopause-related sleep disturbances.

**Data Sources:**

Published studies in English were located by searching PubMed and PsycArticles databases (until September 15, 2022).

**Data Extraction:**

Following full-text review, a final total of 59 articles were included. The search protocol was performed in accordance with PRISMA guidelines.

**Data Analysis:**

A total of 37 studies reported that a nutritional intervention improved some aspect of sleep, and 22 studies observed no benefit. Most (n = 24) studies recruited postmenopausal women, 18 recruited menopausal women, 3 recruited perimenopausal women, and 14 recruited women from multiple groups. The majority of the studies were of low methodological quality. Due to the heterogeneity of the studies, a narrative synthesis without meta-analysis is reported.

**Conclusion:**

Despite the large heterogeneity in the studies and choice of intervention, the majority of the identified studies reported that a nutritional intervention did benefit sleep, and that it is mainly subjective sleep that is improved. More high-quality, adequately powered, randomized controlled trials of the identified nutritional interventions are necessary.

**Systematic Review Registration:**

PROSPERO registration no. CRD42021262367.

## INTRODUCTION

Sleep has a major impact upon a number of health outcomes. Disrupted sleep, or sleep loss, can contribute to a range of deleterious health outcomes, including mortality, obesity, diabetes, and cardiovascular disease.^1^^,^^2^ For this reason, obtaining sufficient high-quality sleep is necessary for maintaining good physical and psychological health.^2^

Although changes to subjective and objective sleep are commonly observed as a function of the normal aging process,^3^ one specific life event that appears to have a direct impact upon sleep is the transition to menopause in women.^4^ Menopause is a complex physiological process and refers to the permanent cessation of menstrual periods due to ovarian follicular depletion, alongside changing hormonal levels of estrogen and progesterone, which can occur naturally or due to surgery, chemotherapy, or radiation.^5^ This typically occurs at approximately 50 years of age for Western women, although the timing can be influenced by lifestyle, race, and ethnicity.^6^

Sleep disturbances are considered to be a core symptom of menopause.^4^ Specific sleep disturbances which are commonly observed during menopause typically include subjective difficulties in falling asleep, awakening too early, excessive daytime sleepiness, and the clinical sleep problem of insomnia disorder (where individuals have difficulties in falling asleep, maintaining sleep, and awakening early, alongside the associated negative daytime consequences of these disturbances).^7^^,^^8^ Clinically, insomnia disorder is more common in women than in men, at a ratio of approximately 2:1,^9^ and the prevalence of insomnia, which is the most commonly observed sleep disorder associated with menopause, increases around the time of menopausal onset.^10^ Also of relevance is the fact that menopause may result in alterations to circadian rhythmicity.^11^ Circadian rhythms refer to the oscillatory rhythms of approximately 24 hours that are displayed by various bodily physiological and behavioral processes (including hormones) and have a direct impact upon sleep timing and quality.^12^^,^^13^ It is well established that aging can affect circadian rhythmicity. For instance, aging affects chronotype, which refers to the preference for the timing of sleep and other daily activities^12^: older adults are more likely to prefer an earlier bedtime, and an earlier morning rise time, compared to young adults.^12^ Additionally, older adults appear to be more sensitive to the effects of a mismatch between an individual’s desired and actual sleep timing (ie, when an individual is awake when their circadian rhythm favors sleep), compared to younger adults^14^; this can impair sleep duration and sleep quality. Aside from the circadian changes that are observed as a function of normal aging, some evidence indicates that, after the menopausal transition, there is a shift toward “morningness,” referring to an individual preference for earlier sleep timing and rise time (relative to “eveningness”)^11^; speculatively, this may be due to changes in hormonal secretion that occur as part of the menopausal transition.^11^

Sleep is a complex physiological process that is typically assessed using various subjective and/or objective measurement methods: subjective methods of sleep measurement can include, for example, questionnaires, estimates of habitual sleep duration, or sleep diaries (sleep logs).^3^ Objective methods of sleep measurement can include actigraphy, which relies on wrist-worn accelerometers to infer sleep and wake patterns based on movement, or polysomnography (PSG).^15^ PSG is the most accurate method of sleep measurement, as this method simultaneously assesses multiple physiological parameters, including overnight brain activity, in order to classify sleep into distinct stages.^16^ The concept of *sleepiness* is also relevant: sleepiness occurs when the brain is forced to transition from a state of arousal to a state of sleep.^17^ Sleepiness is primarily driven by increased sleep pressure caused by extended wakefulness or sleep deprivation, as well as by circadian rhythmicity.^13^^,^^17^ Sleepiness can also be measured subjectively, typically using self-report estimates or questionnaires, or objectively, where PSG is used to quantify sleepiness in the context of a nap opportunity, using the Multiple Sleep Latency Test (MSLT).^16^

It is likely that nutritional interventions and supplements may help to alleviate menopause-related sleep disturbances, since these interventions have been shown to improve relevant aspects of sleep. For instance, relative to placebo, tart cherry juice has been shown to improve objective sleep quantity and quality^18^; tryptophan-rich foods have also been shown to improve subjective and objective sleep quantity and quality.^19^ Therefore, nutritional interventions and supplements are likely to represent one route by which menopause-related sleep disturbances can be treated, or alternatively, used to improve sleep. While a recently published narrative review article has concluded that nutritional interventions may improve menopause-related sleep disturbances,^20^ the main purpose of that previous review was to provide a practical guide for the treatment of menopause-related sleep disturbances through nutritional changes. However, in order to inform the development of nutrition intervention studies with the specific intention of treating sleep disturbances associated with menopause, a systematic search of the literature is necessary, giving consideration to the potential mechanisms by which nutritional interventions can improve specific relevant sleep outcome variables. Therefore, the aim of the present review was to systematically examine the effect of nutritional interventions for menopause-related sleep disturbances, to inform the subsequent development of specific interventional trials.

## METHOD

The search protocol was preregistered in PROSPERO (CRD42021262367) and performed in accordance with PRISMA guidelines.^21^

### Search strategy

Studies published in English were located by searching 2 electronic databases: PubMed (until September 15, 2022) and PsycArticles (from 1967 until September 15, 2022), using the following terms: (“menopause” OR menopause* OR “menopausal” OR “perimenopausal” OR “postmenopausal”) AND (“sleep” OR “insomnia” OR “sleep*” OR “sleep disturbances”) AND (“diet” OR “nutrition” OR nutrition* OR “nutritional intervention” OR “food” OR “dietary intervention” OR “polyphenols” OR “dietary supplements” OR “macronutrients” OR “carbohydrate” OR “fibre” OR “fruit” OR “vegetables” OR “fat” OR “polyunsaturated fatty acids” OR “omega-3” OR “fish oil” OR “fatty acids” OR “unsaturated” OR “shellfish” OR “protein” OR “tofu” OR “legumes” OR “fish” OR “micronutrients” OR “vitamin B12” OR “vitamin D” OR “minerals” OR “magnesium” OR “zinc” OR “niacin” OR “antioxidants” OR “vitamin C” OR “vitamin B1” OR “vitamin B6” OR “folate” OR “phosphorus” OR “iron” OR “selenium” OR “alpha-carotene” OR “calcium” OR “melatonin” OR “tart cherries” OR “phytochemicals” OR “asparagus” OR “tryptophan” OR “amino acid” OR “milk” OR “dairy” OR “kiwifruit” OR “phytoestrogens” OR “isoflavones” OR “soy” OR “strawberries” OR “berry” OR “berries” OR “nuts” OR “whole grains” OR “caffeine” OR “tea” OR “coffee” OR “black cohosh” OR “herbal*”).

#### Eligibility criteria

In the present review, nutritional interventions were defined as specific and measurable changes to diet, with the aim of affecting a relevant outcome measure. These included either specific dietary changes, or the use of nutritional or dietary supplements (in pill, tablet, powder, or liquid form).

Studies were eligible if they specifically assessed the effect of a nutritional intervention, or interventions, upon subjective and objective sleep, where sleep was assessed as either a primary or secondary outcome measure. This included studies where sleep was examined using subjective questionnaire measures, or clinically relevant questionnaire measures. This also included studies where subjective sleep continuity was measured using sleep diaries, from which standard measures of sleep continuity could be derived (eg, subjective sleep efficiency [SE%], number of awakenings [NWAK], wake after sleep onset [WASO], total sleep time [TST], and/or sleep onset latency [SOL]). In addition, studies that incorporated objective measures of sleep continuity (eg, using actigraphy or PSG), and studies that assessed the effects of an intervention upon objective sleep architecture (PSG) were included. Studies that assessed subjective or objective sleepiness (measured using questionnaires or objective methods, such as an MSLT) were also included. The studies were eligible if they included females who were perimenopausal, menopausal, or postmenopausal (as defined by the relevant research study). Unpublished studies and pre-print articles were not specifically sought, but were considered for inclusion if they were relevant, or if they were referenced in eligible studies. The PICOS criteria for the inclusion of studies are listed in Table 1.

**Table 1:** PICOS criteria for inclusion of studies

Parameter	Criterion
Population	Perimenopausal, menopausal, or postmenopausal women
Intervention	Specific dietary changes, or the use of nutritional or dietary supplements (in pill, tablet, powder, or liquid form)
Comparator	Clinical trials with an appropriate control group, or in the case of observational studies, an appropriate pre-intervention/post-intervention comparison
Outcome	Subjective and objective sleep, or subjective and objective sleepiness (assessed as either a primary or secondary outcome measure)
Study design	Observational, experimental, randomized controlled trial (of any design)

Identified articles were excluded if (a) the effect of a nutritional intervention upon sleep could not be evaluated; or if they were: (b) duplicates; (c) review articles; (d) written in a non-English language; (e) conference abstracts; (f) opinion-based letters, or (g) animal studies.

#### Data extraction

Eligible articles were exported to EndNote X9.3 (Clarivate, London, UK), and the abstracts were independently screened against the inclusion criteria by D.P. and G.J.E. using a data extraction form. The methodological quality of studies, in relation to their main aims, was evaluated using the Mixed Methods Appraisal Tool (MMAT).^22^^,^^23^ Each study was assigned a rating from 0 (indicative of a very poor level of methodological quality) to 5 (the highest methodological quality) on the basis of the MMAT. The first 10% of identified papers and the MMAT evaluation was checked by another member of the study team (P.A.-M.).

#### Narrative synthesis

Due to the heterogeneity of studies and methodological approaches, it was not possible to conduct a meta-analysis. Therefore, a narrative description of the identified studies is provided, in line with Synthesis without Meta-analysis (SWiM)^24^ reporting guidelines.

## RESULTS

**Figure 1:**
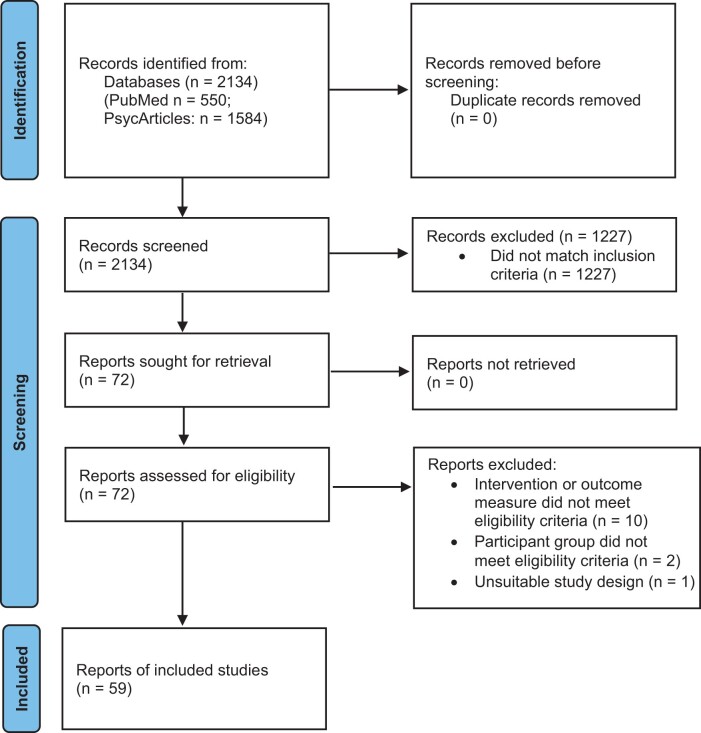
Flowchart of search and selection process.

A total of 2134 potentially relevant articles were identified and screened. No additional results were identified at this point. Following screening, a total of 72 articles were chosen for full-text review. A final total of 59 articles were included (Figure 1).

### Characteristics of studies

There was a large amount of heterogeneity in terms of the choice of nutritional intervention, which is summarized in Table 2.^25–83^ The identified studies also displayed a large amount of heterogeneity regarding the study design, and the results of each study are shown in Table 3.^25–83^ The sample sizes of the identified studies ranged from 18 to 2016 participants and were conducted between 2002 and 2022. The quality of the identified studies ranged from 1 to 5, and the most common MMAT score was 2, which is indicative of a low methodological quality.

**Table 2:** Summary of interventions

Intervention	Number of studies	Number of studies benefiting sleep
Combined nutritional interventions	14^25–38^	10^25^,^28–30^,^32–36^^,38^
Combined herbal formulas	11^39–49^	8^39–41^^,43,44,46–48^
Isoflavones	8^50–57^	4^50^^,^^51^^,^^54^^,^^57^
Soy	5^58–62^	2^58^^,^^61^
Black cohosh (*Cimicifuga racemosa*)	3^63–65^	3^63–65^
Pollen/pollen extract	3^51^^,^^66^^,^^67^	2^51^^,^^67^
Melatonin	2^68^^,^^69^	1^58^
Resveratrol/Trans-resveratrol	2^70^^,^^71^	0
*Disacorea alata* (yam)	1^72^	1^72^
Pomegranate seed oil	1^73^	1^73^
JuicePLUS (no further information provided)	1^74^	1^74^
Jujube seed capsule	1^75^	1^75^
Maca	1^76^	1^76^
Proanthocyanin	1^77^	1^77^
Salvia extract	1^78^	1^78^
Gincosan (*Ginkgo biloba* and P*anax ginseng*)	1^79^	0
*Labisia pumila* var. *alata* extract	1^80^	0
Omega-3	1^81^	0
Pine bark extract	1^82^	0
Probiotic yogurt	1^83^	0

**Table 3:** Summary of identified studies

Reference	Country	Study design	N	Group	Intervention	Sleep measures	Sleep improvement (+, improvement; –, no improvement)	Sleep results	MMAT	Trial registration
**Perimenopausal (n* = * 3)**										
Errichi et al (2011)^82^	Italy	Parallel groups placebo-controlled study	70	Perimenopausal	Pyconogenol: pine bark extract (8 weeks) – 2 × 50 mg tablets with breakfast and dinner	33 menopause symptoms questionnaire (“sleep disorders”)	–	No difference at 8 weeks, relative to placebo	1	Not registered
Kotlarczyk et al (2012)^68^	United States	Randomized placebo-controlled trial	18	Perimenopausal	Melatonin (3 mg) 1 × daily for 1 month	PSQI and average hours slept per month (daily diary)	–	No improvement in PSQI scores or sleep time	1	Not registered
Meissner et al (2006)^76^	Poland	Randomized, placebo-controlled, crossover pilot trial	20	Perimenopausal	Maca-GO: Pre-gelatinized Organic Maca (500 mg) 4 × capsules daily (2 × 30 min before morning meal and 2 × before evening meal) for 4 months	KI: interrupted sleeping pattern	+	Improvement (specific time points not stated)	1	Not registered
**Menopausal (n* *=* * 18)**										
Agosta et al (2011)^25^	Italy	Randomized, controlled, parallel groups trial	634	Menopausal	Estromineral (E) (isoflavones 60 mg, *Lactobaciullus sporogenes*, calcium [5 mg] and vitamin D3 [5 mg]) or Estromineral Serena (ES) (Estromineral and *Magnolia* bark extract [60 mg]) – 1 tablet for 12 weeks	Self-reported presence and severity of insomnia	+	Greater insomnia improvement at weeks 4 and 8 for ES compared with E, expressed as percentage severity reduction	1	Not registered
Davinelli et al (2017)^58^	Italy	Randomized, double-blind, placebo-controlled, trial	60	Menopausal	Fermented soy (1 × 200 mg tablet daily for 12 weeks) containing equol (10 mg) and resveratrol (25 mg)	MRS, NHP (sleep section)	+	Reduced percentage of participants in active group reporting MRS sleep problems at 3 months, relative to placebo, and reduction in individual items of NHP (“take pills to help me sleep”; “I’m waking up in the early hours of the morning”; “I lie awake for most of the night”; “It takes me a long time to get to sleep”; “I sleep badly at night”)	5	ISRCTN: 10128742
De Franciscis et al (2017)^35^	Italy	Controlled	180	Menopausal	ESP: (SI [60 mg], *Lactobillus sporogenes* [109 spores], *Magnolia officinalis* [50 mg], *Vitex agnus-castus* [40 mg], vitamin D [35 µg]; 1 tablet × day for 12 months) C: (Isoflavones [60 mg]; 1 tablet × day for 12 months)	PSQI	+	Reduced PSQI score in ESP compared with C group at 12 months. PSQI subjective sleep quality and latency improvements in ESP group at 12 months compared with C group. Overall improvements at 6 months and 12 months	2	Not registered
De Franciscis et al (2020)^51^	Italy	Prospective observational	164	Menopausal	Pollen extracts (2 tablets per day) or isoflavones [60 mg] for 6 months)	PSQI	+	PSQI reduction in pollen extract and soy group relative to control at both 3 months and 6 months; effect greater in pollen group relative to soy group	3	N/A
Fait et al (2019)^66^	Czech Republic/Slovakia	Prospective observational	104	Menopausal	Pollen extract	MRS (sleep disturbance symptoms)	–	No improvement at 1 month, 2 months, or 3 months relative to before treatment	3	N/A
Guida et al (2021)^63^	Italy	Observational prospective case–control study	163	Menopausal	Isopropanolic extract of *Cimicifuga racemosa* (2 × 20 mg tablet daily [breakfast/dinner] for 3 months)	mMRS (sleep problems)	+	Larger reduction in sleep problem scores (difference between baseline and 3 months) in cases than controls	3	N/A
Mucci et al (2006)^36^	Italy	Randomized, parallel groups trial	89	Menopausal	Soy isoflavones (60 mg), lactobacilli (500 million spores), calcium (141 mg), vitamin D3 (5 µg), *Magnolia* bark extract (60 mg), and magnesium (50 mg) (ES group) or calcium and vitamin D3, 1 tablet per day (Ca+D group) for 24 weeks	Subjective insomnia severity (self-report)	+	Higher percentage absence of insomnia symptoms in ES group relative to Ca+D group at 8 weeks, 12 weeks, and 24 weeks	1	Not registered
Quattrocchi et al (2015)^28^	Italy	Open-label study	151	Menopausal	Phyto complex: (*Trifolium pratense* [200 mg], *Dioscorea villosa* rhizome [100 mg], *Crataegus oxyacantha*; *Griffonia simplicifolia* [60 g], vitamin D3 [3.75 µg], vitamin E [7.5 mg], and zinc gluconate [5 mg]) – 2 tablets per day for first 15 days; 1 tablet per day for 180 days	GCS: insomnia subscale	+	Reduced insomnia at 1-month, 3-month and 6 month follow-up	3	N/A
Russo and Corosu (2003)^31^	Italy	Open-label study	50	Menopausal	Fitomil: Soya isoflavones (80 mg) and *Cimicfuga racemosa* (30 mg)	Insomnia (self-report questionnaire; no further details provided)	–	No difference in insomnia symptoms	2	Not registered
Singhal and Shullai (2016)^55^	India	Open-label study	100	Menopausal	Gabapentin (900 mg) or isoflavones (60 mg) daily for 3 months	PSQI	–	No effect in isoflavones group	2	N/A
Sun et al (2018)^44^	China	Randomized, open-label	390	Menopausal	Heyan Kuntai Capsule (HKC; *Radix rehmanniae*, *Radix paedoniae alba*, *Colla corii asini*, *Rhizoma coptidis*, *Radix scutellariae*, and Poria) – 4 capsules, twice daily for 12 months	Insomnia (measurement tool not specified)	+	Reduced insomnia score at 3-month, 6-month, 9-month and 12-month follow-up, relative to baseline	1	Not registered
Taavoni et al (2013)^38^	Iran	Placebo-controlled trial	100	Menopausal, all with sleep problems (PSQI scores ≥ 5)	Valerian/lemon balm (160 mg/80 mg) 2 × daily capsules. Duration of intervention not stated	PSQI	+	PSQI improvement observed in 36% of intervention group and 8% of placebo. Overall, 5 point deduction in PSQI scores (data not reported)	1	IRCT: 201106302172N10
Terauchi et al (2014)^77^	Japan	Randomized, double-blind, placebo-controlled trial	96	Menopausal	Low-dose (100 mg) or high-dose (200 mg) proanthocyanidin (1 × daily for 8 weeks)	Athens Insomnia Scale (AIS)	+	AIS improvement in high-dose group at 8 weeks relative to baseline	2	Not registered
Vermes (2005)^65^	Hungary	Open-label	2016	Menopausal	Remifemin (black cohosh; dosage not stated) 2 × tablets daily for 12 weeks	KI (insomnia)	+	Slight decrease in insomnia symptom intensity (week 12 vs baseline) – not statistically significant	3	N/A
Villa et al (2017)^34^	Italy	Non-blinded, randomized, parallel group, efficacy study	90	Menopausal	Zemiar: Equol (40%) isoflavones (80 mg), *Passiflora* (178 mg), quercetin (150 mg), resveratrol (10 mg), magnesium (60 mg), calcium (120 mg), vitamin D (5 µg), vitamin K (15 µg); 1 tablet daily × 6 months	KI (insomnia and/or sleep disturbance)	+	Reduced KI insomnia at 6 months relative to baseline	3	Not registered
Wang et al (2017)^46^	China	Open-label	162	Menopausal with poor sleep (PSQI scores >6)	Guizhi Gancao Longgu Muli Tang (combination of 4 traditional Chinese medications: *Ramulus cinnamomi*, Radix Glycyrrhizae Fried, *Os draconis*, and *Concha ostreae*); 400 mL per day (taken twice daily) for 2 weeks	PSQI	+	Reduced PSQI scores 2- and 4-week post-intervention	3	N/A
Xu et al (2021)^47^	China	Retrospective analysis of clinical records	120	Menopausal with insomnia	Chaihu-Guizhi-Longgu-Muli (*Bupleurum*, *Scutellaria*, *Guizhi*, *Codonopsis*, *Ginseng*, Yejiao Teng, raw keel, raw oyster, Poria and Pnellia, rhubarb, wild jujube seed, and ginger), 2 × daily, and *Liuwei Dihuang* (8 pills, 3× per day) for 3 months	PSQI	+	Reduction in PSQI components after 3 months of treatment compared with unspecified “control” group. Effect upon overall PSQI scores not stated	2	Not registered
Yeh et al (2011)^48^	Taiwan	Open-label	67	Menopausal with poor sleep (PSQI scores >6)	Suan Zao Ren Tang (unspecified herbal extract); 3 × 4 g dose daily for 4 weeks	PSQI	+	Reduction in PSQI scores and PSQI components at 1-week and 4-week follow-up relative to baseline	2	N/A
**Postmenopausal (n* *=* * 24)**										
Albert et al (2002)^50^	Spain	Non-randomized, non-placebo-controlled pilot study	190	Postmenopausal	Phyto Soya: Isoflavones (2 × 17.5 mg capsule)	Custom score assessing frequency of nights of bad sleep during previous night	+	Reduction in percentage of “sleep disorder” at 2-month and 4-month follow-up	2	N/A
Auerbach et al (2012)^73^	Austria	Randomized, double-blind, placebo-controlled, trial	81	Postmenopausal	Pomegranate seed oil (2 × 30 mg daily doses for 12 weeks)	MRS II Sleeping Disorders subscale (severity)	+	Reduction in sleeping disorders after 12 weeks	1	EudraCT-NR: 2007-003731-23
Balk et al (2002)^56^	United States	Randomized, double-blind, placebo-controlled trial	27	Postmenopausal	Soy flour and corn cereal (100 mg isoflavones) daily for 6 months	Insomnia severity (4-point scale)		Insomnia severity greater in soy group, relative to placebo, at 6 months	3	Not registered
Duffy et al (2003)^52^	United Kingdom	Double-blind placebo-controlled parallel study	33	Postmenopausal	Soya isoflavone supplement (2 × 30 mg capsules per day for 12 weeks in morning/evening)	SSS, ESS	–	No differences at 12 weeks, relative to placebo	2	Not registered
File et al (2005)^59^	United Kingdom	Double-blind placebo-controlled study	50	Postmenopausal	Soy supplement (50 mg): 1 × capsule (morning) for 6 weeks	ESS	–	No improvement at 6 weeks, relative to placebo	2	Not registered
Hachul et al (2011)^53^	Brazil	Randomized, placebo-controlled, trial	38	Postmenopausal (with insomnia)	80 mg isoflavones and sleep education lecture	Insomnia (KI severity) PSG (2 nights)	–	No KI difference at 2 months or 4 months, relative to placebo. Both groups reported significant reduction in percentage of women reporting moderate/severe insomnia, with significant reduction at 4 months relative to placebo. Increase in SE% in isoflavone group at 4 months, relative to placebo	2	Not registered
Hartley et al (2004)^79^	United Kingdom	Double-blind placebo-controlled	57	Postmenopausal	Gincosan (*Ginkgo biloba* and *Panax ginseng*) – 320 mg/day daily (morning) for 12 weeks	Sleepiness (SSS and ESS)	–	No difference relative to placebo	2	Not registered
Hsu et al (2011)^72^	Taiwan	Randomized, double-blind placebo-controlled trial	50	Postmenopausal	*Disacorea alata* (12 mg) sachet twice daily for 12 months	GCS (insomnia)	+	Reduced insomnia at 6 months and 12 months (intervention group relative to baseline; data not shown)	2	Not registered
Jiang et al (2015)^64^	China	Randomized, double-blind, placebo-controlled, trial	48	Postmenopausal	Black cohosh (20 mg crude drug per tablet) 2 × per day (after meals) for 6 months	Sleep quality (PSQI)/PSG (1 night)	+ (PSG) – (PSQI)	PSG: lower WASO and higher SE% at 6 months relative to placebo. No PSQI improvement	3	Not registered
LeBlanc et al (2015)^27^	United States	Randomized placebo-controlled trial	34 157	Postmenopausal	Elemental calcium carbonate (1000 mg) with vitamin D (400 IU) daily (mean follow-up 5.7 years)	Sleep symptom frequency (waking up several times at night, waking earlier than planned, overall typical sleep pattern, and quality) and sleep disturbance construct (WHIRS)	–	No difference in sleep disturbance at follow-up (5.7 ± 3.2 years)	2	Not registered
Lin et al (2018)^41^	China	Randomized controlled trial	180	Postmenopausal	Ziyin Jianghuo Ningxin Decoction (ZJND; herbal formula comprising 15 herbs), unspecified volume of liquid, taken 2 × daily (am and pm) for 3 months; ZJND and Femoston; Femoston and DHEA; Femoston, ZJND, and DHEA	Unspecified self-report measurement tool: TST, “nighttime sleep time”, WASO, “frequency of WASO”, “longest sleep time,” and “sleep onset time”	+	Femoston, ZJND and DHEA group reported longer “total sleep” and “longer nighttime sleep time” post-treatment (unspecified time point) relative to baseline	1	Not registered
Liu et al (2014)^62^	China	Randomized, placebo-controlled, parallel groups, pilot trial	270	Postmenopausal	40 g of soy flour or 40 g low-fat milk powder + 63 mg of daidzein, daily for 6 months	“Trouble sleeping”	–	No difference between-groups at 6 months relative to placebo	2	NCT01270737
Maffei et al (2022)^57^	Italy	Prospective	71	Postmenopausal	Combined isoflavone compound (isoflavones, *Agnus castus*, and *Magnolia* extracts) – 1 tablet at bedtime for 12 months	ISI	+	ISI scores reduced at 12 months	3	NCT03699150
Mahmoudi et al (2020)^75^	Iran	Randomized, double-blind, placebo-controlled trial	106	Postmenopausal with poor sleep (PSQI scores ≥ 5)	Jujube seed capsule (250 mg) twice a day for 21 days	PSQI	+	PSQI scores decreased post-intervention (relative to baseline and compared with control group)	2	Not registered
Plotnikoff et al (2011)^42^	United States	Randomized, double-blind, placebo-controlled, trial	178	Postmenopausal (with hot flashes)	TU-025 (keishibukuryogan): 7.5 g or 12.5 g per day for 12 weeks	PSQI	–	No effect upon sleep quality at 12 weeks	2	NCT00119418
Purzand et al (2020)^61^	Iran	Randomized, double-blind, placebo-controlled, trial	180	Postmenopausal	Soybean (Soygan 500 mg capsule), omega-3 fatty acids (Omega-rex 1000 mg soft gel) daily for 12 weeks	MRS	+	Highest reduction (mean difference) in sleep problems for soybean group (–1.15). Reduction in omega-rex group (–.84)	4	IRCT: 20200222046584N1
Rattanatantikul et al (2022)^29^	Thailand	Randomized, double-blind, placebo-controlled, trial	110	Postmenopausal	Estosalus: isoflavones (100 mg), black cohosh (520 mg), chasteberry (400 mg), and evening primrose oil (500 mg) – 1 × capsule (1000 mg) daily (after breakfast) for 12 weeks	MRS (sleep problems): categorical: not severe/severe	+	Reduced proportion of severe sleep problems at 6 weeks and 12 weeks in treatment group, compared with placebo	4	TCTR: 20190417001
Shafie et al (2022)^83^	Iran	Randomized, triple-blind, placebo-controlled, trial	66	Postmenopausal	Probiotic yogurt (100 g) daily for 6 weeks	PSQI	–	No difference in PSQI scores at 6 weeks	2	IRCT: 20120718010324N57
Siriyong et al (2021)^43^	Thailand	Open-label	35	Postmenopausal	Thai herbal formulations (90 mL, 3 × daily for 4 weeks)	MRS (sleep problem) and PSQI	+	Reduced MRS sleep problem and PSQI scores at 4-week and 8-week follow-ups	2	N/A
Sun (2003)^32^	United States	Open-label study	72	Postmenopausal	Morning Menopause Formula: *Panax ginseng* (50 mg), black cohosh (20 mg), soy (100 mg), and green tea extract (250 mg) Evening Menopause Formula: black cohosh (20 mg), soy (100 mg), kava (200 mg), hops (100 mg), and valerian extracts (200 mg) 1 × morning and 1 × evening capsule for 2 months	Sleep quality (PSQI) and subjective number of awakenings (1-day diary)	+	Reduced number of awakenings at week 4 and week 8 relative to baseline. Reduced total PSQI scores at week 4 and week 8, and in all PSQI components except sleep medication	3	N/A
Thaung Zaw et al (2020)^70^	Australia	Randomized, double-blind, placebo-controlled crossover trial	125	Postmenopausal	Resveratrol (75 mg) 2 × capsules (morning and evening) for 12 months	PSQI	–	No statistical difference (% of participants showing poor sleep: PSQI scores ≥ 5). Mean PSQI scores not reported	4	ACTRN: 12616000679482p
Walecka-Kapica (2014)^69^	Poland	Open-label	81	Postmenopausal	Melatonin (5 mg) and standardized diet (1500 kcal/day) for 24 weeks	Modified ISI (quality of life item replaced with assessment of shortening of sleeping time)	+	Reduced ISI scores at 24 weeks	3	N/A
Wong et al (2017)^71^	Australia	Randomized, double-blind, placebo-controlled trial	80	Postmenopausal	Trans-resveratrol (75 mg) 2 × daily for 14 weeks	PSQI	–	No change in PSQI total or component scores relative to baseline, expressed as percentages (mean values not shown)	1	ACTRN: 12615000291583
Zeidabadi et al (2020)^78^	Iran	Randomized, double-blind, placebo-controlled, trial	66	Postmenopausal	*Salvia officinalis* extract; 3 × 100 mg tablets daily for 3 months	MRS (sleep problems) and PSQI	+	Reduced MRS and PSQI scores in intervention group at 2-month follow-up	2	Not registered
**Women from multiple groups (n* *=* *14)**										
Chang et al (2012)^39^	United States/South Korea	Randomized, double-blind placebo-controlled trial	64	Premenopausal, perimenopausal, and postmenopausal	EstroG-100: (*Cynachum wilfordii*, *Phlomis umbrosa*, and *Angelica gigas* [257.05 mg]) – 1 tablet, twice a day, for 12 weeks	KI (insomnia)	+	Reduced insomnia scores at 6-week and 12-week follow-up relative to placebo	3	ISRCTN: 95953457
Chinnappan et al (2020)^26^	Canada	Randomized, double-blind, placebo-controlled, trial	119	Perimenopausal and menopausal	Herbal formulation (Nu-femme): 200 mg *Labisia pumila* (SLP+) and 50 mg *Eurycoma longifolia* (Physta); 2 × capsules daily for 24 weeks	Not stated (MRS or MENQOL)	–	No differences relative to placebo (12 weeks or 24 weeks)	3	Not registered
Cohen et al (2014)^81^	United States	Randomized controlled trial	355	Perimenopausal and menopausal	Omega-3 (1.3 g daily for 12 weeks) and simultaneous exercise or usual physical activity	ISI, PSQI	–	No differences in ISI or PSQI at 12 weeks	5	Not registered
Frigo et al (2021)^37^	Brazil	Randomized placebo-controlled single-blind trial	48	Pre-menopausal, perimenopausal, and postmenopausal	Soybean (80.73 mg) and flaxseed phytoestrogen cereal bar (1 per day for 90 days)	Insomnia (KI)	–	No difference at 90 days, relative to placebo	2	ReBEC: 6z8qqy
Hirose et al (2016)^54^	Japan	Randomized, double-blind, placebo-controlled, trial	90	Pre-menopausal, perimenopausal, and menopausal (surgically induced)	Ultra-low-dose (12.5 mg) or low-dose (25 mg) isoflavone aglycone daily for 8 weeks	Insomnia (AIS)	+	Larger AIS score reduction in low dose group at 8 weeks, relative to placebo	3	UMIN-CTR: 000011876
Hirose et al (2018)^60^	Japan	Randomized, double-blind, placebo-controlled	96	Pre-menopausal, perimenopausal, and postmenopausal, all with fatigue	Low-dose (600 mg) or high-dose (1200 mg) soy lecithin tablets (6 × daily after breakfast) for 8 weeks	Objective sleep: actigraphy	–	No effect upon actigraphy (TST, SL, SE%, or zero-crossing or metabolic equivalent during awake and sleep phase, daily variation in sleep time, or episodes of nocturnal awakening)	2	UMIN-CTR: 000017127
Lai et al (2005)^40^	Taiwan	Prospective observational	126	Perimenopausal and postmenopausal (with hot flashes)	TMN-1 (mixture of 21 plant species used in commercially available traditional Chinese medicines); 4 g granules, 3 times per day, for 12 weeks	KI (sleep disturbances)	+	Higher odds of insomnia improvement at follow-up (week 4 and week 12)	3	N/A
Lello et al (2021)^67^	Italy	Prospective observational	108	Perimenopausal and postmenopausal	Purified Cytoplasm of Pollen (dosage and frequency not stated)	GCS (difficulty falling asleep)	+	Reduction in median GCS score at 3 month follow-up	1	N/A
Norhayati et al (2014)^80^	Malaysia	Randomized, double-blind, placebo-controlled, parallel group trial	197	Premenopausal and postmenopausal	*Labisia pumila var alata* extract (40 mg) – 5 capsules, 2 × daily (am and pm) for 16 weeks	WHQ (Sleep problems)	–	No significant change at 16 weeks	2	Not registered
Rotem and Kaplan (2007)^30^	Israel	Randomized, double-blind, placebo-controlled, trial	50	Premenopausal and postmenopausal	Phyto-Female Complex: black cohosh (100 mg), dong quai (75 mg), milk thistle (75 mg), red clover (50 mg), American ginense (50 mg), and chaste-tree berry (50 mg); 2 × capsule daily for 3 months	Subjective sleep quality (rated 1 to 5; where 1 was highest)	+	Better sleep quality score at end of treatment in intervention group, relative to placebo	2	Not registered
Siebler et al (2016)^74^	Germany	Open-label study	28	Perimenopausal and postmenopausal	JuicePLUS*+* (further information not given)	Subjective sleep problems (MRS)	+	Reduction in percentage of sleep problems at 16 weeks	1	N/A
Terauchi et al (2011)^33^	Japan	Retrospective analysis of clinical records	151	Perimenopausal and postmenopausal, all with sleep problems	Tokishakuyakusan (TJ-23; Tangkuei and peony powder), Kamishoyosan (TJ-24; Augmented Rambling Powder) or Keishibukuryogan (TJ-25; Cinnamon twig and Poria pill) – 7.5 g daily, frequency not provided	Subjective sleep disturbance score, subjective sleep quality (sleep duration [h], sleep onset ease, number of awakenings per night, and sleep satisfaction)	+	Reduced sleep disturbance score at follow-up (5 months) in TJ-24 and TJ-25 groups relative to control (health/nutrition education). No difference in sleep duration or disrupted sleep	1	N/A
Terauchi et al (2011)^45^	Japan	Retrospective analysis of clinical records	77	Perimenopausal and postmenopausal	TJ-25: (keishibukuryogan) – 7.5 g daily	Subjective sleep quality and satisfaction	–	No change in percentages of participants reporting “difficulty in initiating sleep” or “nonrestorative sleep” 6 months post-intervention	2	N/A
Zhang et al (2020)^49^	China	Randomized, single-blind, placebo-controlled, trial	98	Perimenopausal and postmenopausal	Gengnianchun (herbal formula) one 11.6 g sachet of granules daily for 12 weeks	PSQI	–	No effect at 4- week, 8- week, or 12-week follow-up	2	ChiCTR: IOR-17012903

*Abbreviations:* ACTRN, Australian New Zealand Clinical Trials Registry; AIS, Athens Insomnia Scale; ChiCTR, Chinese Clinical Trial Registry; DHEA, Dehydroepiandrosterone; ESS, Epworth Sleepiness Scale; GCS, Greene Climacteric Scale; HKC, Heyan Kuntai Capsule; IRCT, Iranian Registry of Clinical Trials; ISI, Insomnia Severity Index; ISRCTN, International Standard Randomised Controlled Trial Number; KI, Kupperman Index; MENQOL, Menopause-Specific Quality of Life; MMAT, Mixed Methods Appraisal Tool; mMRS, Menopause Rating Scale (modified); MRS, Menopause Rating Scale; N/A, not applicable; NHP, Nottingham Health Profile; PSG, polysomnography; PSQI, Pittsburgh Sleep Quality Index; ReBEC, Registro Brasileiro de Ensaios Clínicos (Brazilian Clinical Trials Registry); SE%, sleep efficiency; SI, soy isoflavones; SL, Sleep Latency; SSS, Stanford Sleepiness Scale; TCTR, Thai Clinical Trials Registry; TST, Total Sleep Time; UMIN-CTR, University Hospital Medical Information Network Clinical Trials Registry; WASO, Wake After Sleep Onset; WHIRS, Women’s Health Initiative Insomnia Rating Scale; WHQ, Women’s Health Questionnaire; ZJND, Ziyin Jianghuo Ningxin Decoction.

A total of 3 studies recruited perimenopausal women,^68^^,^^76^^,^^82^ 18 recruited menopausal women^25,28,31^^,^^34–36^^,^^38^^,^^44^^,^^46–48^^,^^51^^,^^55^^,^^58^^,^^63^^,^^65^^,^^66^^,^^77^ and a total of 24 primarily recruited postmenopausal women.^27,29,32^^,^^41–43^^,50,52,53,56,57,59,61,62,64,^^69–73^^,75,78,79,83^ Fourteen studies recruited women from multiple groups.^26^^,^^30^^,^^33^^,^^37^^,^^39^^,^^40^^,^^45^^,^^49^^,^^54^^,^^60^^,^^67^^,^^74^^,^^80^^,^^81^ Only 6 studies specifically assessed the impact of interventions upon women with self-reported sleep disturbances,^33,38^,^46–48^^,75^ 2 of which involved a retrospective analysis of clinical records.^33^^,^^47^ The majority of studies assessed the effect of the interventions upon sleep by measuring the impact upon sleep-related symptoms of menopausal symptom questionnaire measures.^27–29^^,^^34^^,^^37^^,^^39^^,^^40^^,^^43^^,^^53^^,^^56^^,^^58^^,^^61–63^^,^^65–67^^,^^72–74^^,^^76^^,^^78^^,^^80^^,^^82^ Another common outcome measure involved examining post-intervention sleep quality, or subjective insomnia symptom severity, using established questionnaire measures which were developed specifically for that purpose (eg, the Pittsburgh Sleep Quality Index [PSQI] or the Insomnia Severity Index [ISI]).^32,35,38,42,43^,^46–49^^,51,54,55,57,64,^^68–71^^,75,77,81,83^ Additionally, 6 other studies reported the subjective impact upon sleep, but established questionnaire measures were not used.^25^^,^^27^^,^^36^^,^^44^^,^^45^^,^^50^ For instance, 2 studies used the self-reported presence and severity of insomnia^25^^,^^36^; 1 reported the frequency of self-reported sleep disturbances (eg, how often nocturnal awakenings occurred in a previous time frame)^27^; or a custom score that assessed the frequency of perceived nights of “bad sleep”, as reported in^50^; One study used an unspecified insomnia disorder measurement tool^44^; and 1 study used an unspecified measure of subjective sleep quality and satisfaction.^45^

Only 4 studies concentrated on the impact of any intervention upon subjective sleep continuity measures.^32^^,^^33^^,^^41^^,^^68^ These included the average number of hours slept per month (derived from a daily diary),^68^ the subjective number of awakenings (also derived from a diary),^32^ and, in one study, where a sleep disturbance score was calculated, alongside subjective sleep quality (in terms of sleep duration, the ease of sleep onset, the number of awakenings per night, and perceived satisfaction with sleep).^33^ A further study used an unspecified self-report measure that assessed total sleep time, wake after sleep onset, and other non-standard sleep continuity parameters (such as “frequency of wake after sleep onset” and “longest sleep time”).^41^ Three studies measured the impact upon subjective sleepiness, by using established questionnaire measures, including the Stanford Sleepiness Scale and the Epworth Sleepiness Scale.^52^^,^^59^^,^^79^ Finally, only 3 studies measured the effect of any intervention upon objective sleep: 1 study used actigraphy as the main outcome^60^ and 2 studies used 1 or 2 nights of PSG, respectively.^53^^,^^64^

Overall, a total of 37 studies reported that the nutritional intervention improved some aspect of sleep,^25^^,^^28–30^^,^^32–36^^,^^38–41^^,43,44,^^46–48^^,50,51,53,54,^^56–58^^,61,63,64,67,69,^^72–78^ and a total of 22 studies observed no benefit upon sleep, or sleepiness.^26,27,31,37,42,45,49,52,55,59,60,62,65,66,68,70,71^^,^^79–83^ Of the 37 studies that showed a positive effect upon sleep, this included a total of 18 studies where improvements were observed using a menopause symptom questionnaire, such as the Greene Climacteric Scale (GCS), Menopause Rating Scale (MRS), or Kupperman Index (KI), which assessed some element of sleep such as the presence or absence of sleep disorders.^28^^,^^29^^,^^34^^,^^36^^,^^39^^,^^40^^,^^43^^,^^58^^,^^61^^,^^63^^,^^65^^,^^67^^,^^72–74^^,^^76^^,^^78^^,^^82^ These 37 studies also included a total of 10 studies in which a benefit upon subjective sleep quality was observed, as measured using a validated and commonly used measure of sleep quality (PSQI).^32^^,^^35^^,^^38^^,^^46–49^^,^^51^^,^^75^^,^^78^ Four studies found that nutritional interventions reduced the severity of the sleep disorder insomnia,^54^^,^^57^^,^^69^^,^^77^ as measured using established questionnaires including the Athens Insomnia Scale (AIS)^54^^,^^77^ and the standard or modified ISI.^57^^,^^69^ Only 1 study reported an improvement in subjective sleep continuity, which was measured using a 1-day sleep diary.^32^ A total of 6 studies found improvements when subjective sleep was assessed using unspecified, or custom, measures of assessment.^25^^,^^30^^,^^33^^,^^41^^,^^44^^,^^50^ This included studies using self-report measures of insomnia^25^^,^^44^ or self-report assessment of the frequency of nights of bad sleep during the previous night^50^, or subjective sleep quality.^30^ Other studies have found a benefit upon unspecified measures of sleep quality and quantity,^33^ or subjective measurement of sleep continuity which has included standard continuity variables such as total sleep time or wake after sleep onset (WASO), in addition to custom variables including “nighttime sleep time”, “frequency of WASO”, “longest sleep time” and “sleep onset time”.^41^ Finally, only 1 study specifically examined the impact upon objective sleep, which was measured using PSG. In this study, the intervention (black cohosh) resulted in lower WASO and higher SE%, which represents a reduction in nocturnal wake duration and increased sleep quality respectively, relative to placebo; however, no corresponding benefit to subjective sleep quality (PSQI) was observed.^64^

Overall, positive results were observed in 1 study involving only perimenopausal women,^76^ in 15 studies involving menopausal women,^25^^,^^28^^,^^34–36^^,^^38^^,^^44^^,^^46–48^^,^^51^^,^^58^^,^^63^^,^^65^^,^^77^ in 14 studies involving postmenopausal women,^29^^,^^32^^,^^41^^,^^43^^,^^50^^,^^57^^,^^61^^,^^64^^,^^69^^,^^71–73^^,^^75^^,^^78^ and in 7 studies involving women from multiple groups.^30^^,^^33^^,^^39^^,^^40^^,^^54^^,^^67^^,^^74^ The studies are summarized in more detail in Table 3.

## DISCUSSION

Despite the heterogeneity of the identified studies, and in the choice of intervention (Table 2), the majority of the studies reported that a nutritional intervention did benefit sleep. While these results primarily indicate that it is subjective sleep that is improved by nutritional interventions, interestingly, 2 studies are also suggestive of improvements to objectively measured sleep, which was assessed using PSG.^53^^,^^64^ The limitations of the identified studies, the implications of the studies that have observed positive effects, and potential directions for future nutritional trials aimed at alleviating or preventing menopausal-related sleep disturbances are summarized below.

### Promising nutritional interventions

A number of identified studies reported positive results,^25,28–30,32–36,38–41,43,44,46–48,50,51,53,54,56–58,61,63,64,67,69,72–78^ and despite the heterogeneity in the interventions that have been used, there are several specific interventions that do appear to be worthy of further investigation.

Isoflavones, either used as a standalone intervention, or in combination with other agents, appear to be particularly promising, as benefits have been found upon subjective sleep.^25^^,^^29^^,^^36^^,^^50^^,^^53^^,^^54^^,^^56^^,^^57^ However, it should be noted that not all studies were positive, including 1 study that did not observe an effect upon sleepiness.^51^^,^^53–55^ The dosage may influence the degree of the therapeutic effect, as 1 study has found that a higher dose (25 mg daily) was more effective than a lower dose (12.5 mg) in the treatment of self-reported insomnia symptoms.^54^ In terms of other interventions, 3 studies indicated that soy or soybean-based interventions were beneficial in terms of subjective sleep^35^^,^^58^^,^^61^; the effects of soy are typically attributed to their high isoflavone content.^84^

Additionally, black cohosh may warrant further investigation: in 1 study it was found to improve subjective sleep problems (assessed on the basis of a menopausal symptom assessment),^63^ and in another study, it improved objective sleep, in terms of PSG-assessed markers of nocturnal wake duration (WASO) and objective sleep quality (SE%), although not subjective sleep quality, relative to placebo, at six months follow-up.^64^

Although other interventions, including pomegranate seed oil,^73^ pollen extracts,^51^ organic Maca,^76^ jujube seed capsule,^75^ and salvia extract,^78^ appear to benefit subjective sleep, and high doses of proanthocyanin may benefit subjective insomnia symptoms,^77^ in all cases, only 1 study has assessed the impact of each of these interventions. A variety of other studies have indicated that combined nutritional products benefit subjective self-reported insomnia, sleep quality, sleep disturbances, and nocturnal awakenings,^28–30^^,^^32–34^^,^^36^^,^^74^ and similarly, the use of melatonin, combined with a reduced caloric intake, reduced subjective insomnia severity.^69^ Finally, only 5 studies have specifically focused on the use of nutritional interventions in women with clinically significant poor sleep quality, and insomnia, respectively.^38^^,^^46–48^^,^^53^ In the first study, valerian and lemon balm improved subjective sleep quality in women with poor sleep quality,^38^ which was defined as PSQI scores of ≥5, which is an accepted cut-off value for poor sleep. However, in this study, the duration of the valerian and lemon balm usage was not stated.^38^ Similarly, in another study, jujube seed capsules improved subjective sleep quality (expressed as reduced PSQI scores) at a 21-day follow-up time point in women with baseline PSQI scores of ≥5.^75^ Finally, in women with PSQI scores of >6, a combination of 4 traditional Chinese medications (Guizhi Gancao Longgu Muli Tang) improved sleep quality at follow-up.^48^ In postmenopausal women with self-reported insomnia, isoflavones benefited sleep,^53^ and another study found that a combination of herbal ingredients reduced PSQI scores at follow-up, although this was compared to an unspecified control group, and details regarding the PSQI scores were not stated.^47^ However, in both studies, the definition of insomnia was not clearly stated; consequently, it is not clear whether women with insomnia symptoms were recruited, or if participants had an appropriate clinical diagnosis of insomnia disorder.

Overall, despite the heterogeneity in the interventions, and the relatively low number of studies, the most consistent finding is that isoflavone-based interventions do appear to benefit subjective sleep,^25^^,^^29^^,^^36^^,^^50^^,^^53^^,^^54^^,^^56^^,^^57^ and, as mentioned, 1 study has indicated that there might be a dose–response effect upon sleep.^54^ That said, one limitation is that the precise mechanism by which isoflavones could affect sleep is currently unclear and will require further investigation.^85^ One speculative possibility is that, because isoflavones are phytoestrogens, which bind to estrogen receptors, and as isoflavones may reduce menopausal symptoms,^86^ isoflavones may improve sleep by alleviating or improving menopausal symptom severity. This does appear to be a plausible explanation, since it is known that sleep disturbances are associated with menopausal symptoms.^7^^,^^8^

Black cohosh also appears to improve subjective and objective sleep^63–65^; however, as with isoflavones, the exact mechanism of action for this intervention upon sleep is not well established. However, it is possible that black cohosh can affect the neurotransmitters that modulate sleep/wake regulation, including serotonin (5-HT) and γ-aminobutyric acid (GABA).^64^ Although only 3 studies have assessed the impact of this intervention, the results of all 3 were positive, and this intervention is certainly likely to be worthy of further investigation in the context of a larger-scale trial. Finally, as stated, various other specific interventions show promise in terms of benefitting subjective sleep^51^^,^^73^^,^^75–78^; however, to date, only 1 study of each of these interventions has shown that there is a benefit. Overall, larger, higher-quality studies need to be conducted to replicate these findings.

### Nutritional interventions that do not benefit sleep

It should also be noted that a number of the identified studies have demonstrated that a range of nutritional interventions do not benefit sleep or sleepiness (Table 2). However, for some interventions, only 1 or 2 studies investigating the subsequent effect upon sleep have been conducted. For instance, this was the case in studies that investigated resveratrol and trans-resveratrol,^70^^,^^71^ Gincosan,^79^  *Labisia pumila* var. *alata* water extract,^80^ pine bark extract,^82^ probiotic yogurt,^83^ and omega-3.^81^ As was the case with the nutritional interventions that did observe a positive effect upon sleep, there was a great deal of heterogeneity between studies in terms of the choice of intervention, the chosen outcome measure, and the menopause status of participants. It is likely that the primary reason for these null results is due to such a wide range of methodological limitations. Although these specific limitations will be discussed in detail in the following section, they have included problems such as lack of trial preregistration, poor methodological quality, and in the choice and suitability of the sleep outcome measure.

Despite the apparent heterogeneity in the negative studies, of the studies where no benefit was observed at all, there are 2 main similarities. Firstly, sleep or sleepiness was not considered to be the primary outcome measure,^70^^,^^71^^,^^79–82^ and, notably, in 3 studies the primary aim was to demonstrate the efficacy and safety, or efficacy and tolerability, of the intervention,^80–82^ rather than to assess the clinical effectiveness upon a pre-specified primary outcome. Secondly, these studies did not provide an underlying mechanistic explanation to justify an expected positive effect upon sleep; in these studies, sleep was generally considered as part of an overall menopausal symptom profile.^70^^,^^71^^,^^79–82^ Overall, as the focus of these studies was to only assess the impact upon sleep as part of a menopausal symptom profile (as a secondary measure), and as there was no sound mechanistic justification for an expected benefit to sleep, it is perhaps unsurprising that no benefit was observed. As an additional point, the majority of the null studies recruited only postmenopausal women,^70^^,^^71^^,^^79^^,^^83^ and included studies in which the intervention was Gincosan,^79^ probiotic yogurt,^83^ or resveratrol and trans-resveratrol.^70^^,^^71^ Despite the apparent lack of impact of the interventions, this does perhaps suggest that future work should consider if menopausal status is likely to have an impact upon the effectiveness of a given intervention.

### Limitations of identified studies

Despite the fact that a diverse range of nutritional interventions do appear to benefit menopause-related sleep disturbances, the identified studies have a number of limitations that should be considered and addressed.

First, overall, most of the studies identified in the present review were of a poor methodological quality; additionally, many of the studies identified stated that the primary aim of their study was to demonstrate the efficacy and safety of their chosen nutritional intervention, and not necessarily the effectiveness.^35^^,^^36^^,^^40^^,^^48–50^^,^^80^ This was the case irrespective of whether positive or null effects were observed. Similarly, the majority of all identified studies only examined the impact of nutritional interventions upon sleep as a secondary outcome,^26^^,^^45^^,^^52^^,^^59^^,^^62^^,^^63^^,^^72^^,^^73^^,^^79^^,^^81^ or measured the effect upon sleep within the aim to assess the impact of the nutritional interventions upon various menopausal symptoms more generally.^29–31^^,^^39^^,^^41–44^^,^^54^^,^^57^^,^^67^^,^^78^ Another potential problem is that many of the clinical trials that were identified in the present review did not appear to have been preregistered.^25–27^^,^^30^^,^^31^^,^^34^^,^^36^^,^^41^^,^^44^^,^^52^^,^^53^^,^^56^^,^^59^^,^^64^^,^^68^^,^^72^^,^^75–77^^,^^79–82^ The lack of preregistration is problematic, as the preregistration of clinical trials is necessary to ensure transparency, and to also minimize the impact of, or prevent, publication bias and the selective reporting of results upon trial completion.^87^ In some studies, full details regarding the dosage, contents, or the frequency of usage of each intervention was not stated,^33^^,^^41^^,^^48^^,^^65–67^ which is likely to hinder replication studies.

Another problem is that the majority of the identified research studies used combined interventions, where the specific intervention or supplement was comprised of multiple potentially “active” ingredients.^25–36^^,^^39–41^^,^^43^^,^^44^^,^^46^^,^^47^^,^^57^ A clear limitation of this approach is that, generally, a sound rationale for the specific choice of dosage and specific combination of the intervention was not provided when the results were reported; in some instances, these combined multiple “active” ingredients that can affect sleep in their own right. For instance, in 1 study, participants were given a morning menopause formula consisting of *Panax ginseng*, black cohosh, soy, and green tea extract, and an evening menopause formula consisting of black cohosh, soy, kava, hops, and valerian extracts.^32^ Previous work has demonstrated that l-theanine, which is an amino acid contained within tea, may enhance GABA levels; GABA has a key role in the regulation of sleep and wake^88^^,^^89^; additionally, valerian is a sleep-promoting medication, potentially due to the lignan derivate olivil binding to adenosine A1 and GABA-A receptors, subsequently resulting in sedative effects.^90^ Consideration should be given as to whether or not such combinations of ingredients or compounds within the interventional product have the potential to cause additive or synergistic effects, since this is very likely to affect sleep. Finally, while 1 study reported that a proprietary intervention (“JuicePLUS+”) was found to be effective in terms of reducing subjective sleep problems at follow-up, there was no description of what was contained in the intervention in the study report^74^; therefore, the underlying mechanism of action is not clear. When proprietary interventions are used, the contents of the intervention should be clearly stated, and the proposed mechanism of action should be fully explained and justified.

A further issue is that, when nonpharmacological interventions are used alongside nutritional interventions, this may result in additive effects, where the impact of the nutritional intervention alone is not well-understood. This was the case in 2 of the identified studies, in which it was not possible to isolate the effects of the nutritional intervention from the effect of the additional interventions that were used at the same time.^53^^,^^81^ In 1 study, omega-3 was administered alongside a placebo, but the participants were concurrently randomized to receive yoga, exercise, or usual physical activity^81^; although there was no difference between the omega-3 and placebo groups at the 12 weeks follow-up, importantly, both groups showed improved sleep quality, and reduced insomnia severity.^81^ While mechanistically there is reason to support the administration of omega-3, due to its potential role within the melatonin-producing pineal gland, which is a key region involved in sleep/wake regulation,^91^^,^^92^ meditative movement interventions such as yoga, and physical activity, can also both affect sleep,^93^^,^^94^ and this may have masked the true effect of omega-3 upon sleep.

Similarly, another study investigated the effects of isoflavones in menopausal women with insomnia disorder^53^ and although participants received a sleep education lecture at the beginning of the study, which summarized sleep hygiene, menopausal symptoms and general healthcare, sleep education has been shown to result in improvements to sleep in its own right.^95^ This study observed that whilst there was no difference in insomnia at the 2-month or 4-month follow-up time points, relative to a placebo condition, objective SE% improved in the isoflavones group, and importantly, both groups showed a significant reduction in the percentage of women reporting moderate or severe insomnia at 4 months follow-up.^53^ Given that it is possible that, for instance, both exercise and sleep hygiene can influence sleep, comparator conditions need to be chosen with caution.

One final issue is in regard to the choice of outcome measures. As stated, many studies focused upon sleep as a secondary outcome,^26^^,^^45^^,^^52^^,^^59^^,^^62^^,^^63^^,^^72^^,^^73^^,^^79^^,^^81^ or as part of various menopausal symptoms.^29–31^^,^^39^^,^^41–44^^,^^54^^,^^57^^,^^67^^,^^78^ However, an issue is that many studies assessed whether or not the intervention was effective simply by reporting the percentage of study participants who reported subjective improvements, or the absence of symptoms at a follow-up time point, and not by comparing subjective or objective numerical improvements using an appropriate statistical test.^29^^,^^50^^,^^58^^,^^71^^,^^74^ Similarly, other studies simply directly compared intervention and non-intervention groups at follow-up time points in order to judge the effectiveness of the interventions, without incorporating baseline assessments from both groups into the statistical tests^62^^,^^64^; this is problematic and may contribute to the reason as to why many of the studies observed null results.^26^^,^^27^^,^^31^^,^^37^^,^^52^^,^^55^^,^^59^^,^^60^^,^^62^^,^^65^^,^^66^^,^^68^^,^^70^^,^^71^^,^^79^^,^^81^^,^^82^ To accurately determine whether or not a given intervention has a beneficial effect upon sleep, future studies should use appropriate statistical tests and clearly report accompanying measures of effect size.

### Suggestions for future research

Overall, these results clearly indicate that high-quality, adequately powered, randomized controlled trials of nutritional interventions are necessary for research into the treatment of menopause-related sleep disturbances. Given that qualitatively, the majority of the studies that were identified in the present review were of a poor methodological quality, future trials need to have clear, well-defined, and appropriate sleep outcome measures.

In terms of specific future research directions, aside from the high-quality trials of the specific interventions identified in the previous section, perhaps one of most important is that ideally, nutritional interventions should have a sound underlying mechanistic justification in relation to the target symptom (eg, valerian may be sleep-promoting due to its impact upon adenosine A1 and GABA-A receptors^90^). It is perhaps surprising that interventions which have been shown to improve sleep in other groups have not yet been trialled in relation to menopause-related sleep disturbances, such as tart cherry juice,^18^ which does appear to improve objective sleep and promote exogenous melatonin levels, or tryptophan-rich foods.^19^ These could potentially be trialed in the short term.

Future studies should also consider that the pathophysiology of sleep disturbances (eg, the disruption to sleep) in relation to menopause is complex, and this should inform the choice of design and participant group. First, sleep disturbances can occur because of menopause, but they can also occur as a secondary cause: physiological or psychological changes that happen alongside menopause, such as vasomotor symptoms, depression, and/or anxiety,^7^^,^^96^ trigger the sleep disturbances. For instance, the vasomotor symptom of hot flashes is associated with sleep disturbances in menopausal women.^10^ Although these effects may be difficult to disentangle due to the bidirectional associations between, for example, sleep, anxiety, and depression,^97–100^ well-designed studies which carefully consider the nature and time course of the sleep disturbance in participants (eg, primary vs secondary) may be able to more accurately determine if the chosen intervention is effective. Finally, future studies should consider methodological design choices such as follow-up duration, or the dosage, in order to assess and optimize both the short-term and long-term effectiveness of interventions. For instance, from the identified studies, it is likely that the dosage of the intervention may have an impact upon the subsequent effectiveness.^54^

Future trials should also use both subjective and objective measures of sleep as outcome measures. Although subjective sleep quality can be assessed using questionnaires such as the PSQI,^101^ subjective sleep diaries can also be used to assess sleep; these involve individuals recording the times at which they sleep and wake up, sleep duration, and the frequency and duration of nocturnal awakenings, typically for a minimum period of 1 week.^102^ Particular advantages of sleep diaries are that they are cheap and easy to use, and that measures of sleep continuity can easily be derived from completed diary entries (eg, total sleep time, time in bed, and SE%; the latter is a marker of subjective sleep quality)^3^; this is importantas insomnia disorder is diagnosed based on the subjective experience of sleep, and insomnia disorder is common in menopause.^7^ One potential advantage of sleep diaries is that they can indicate which particular aspect of sleep continuity is improved by the intervention; this would researchers to determine if, for example, an intervention reduces the frequency and duration of nocturnal awakenings, or if it improves SE%. Similarly, where studies intend to assess the impact of an intervention upon a specific sleep disorder (eg, insomnia disorder), care must be taken to ensure that participants are recruited in accordance with recognized diagnostic criteria, such as those contained within the Diagnostic and Statistical Manual of Mental Disorders, or the International Classification of Sleep Disorders.^103^^,^^104^

Actigraphy could also be employed as a marker of objective sleep, since this is generally considered to be the most suitable alternative to PSG when used for these purposes.^15^ Despite the relatively low cost and ease of use of this technique, it is perhaps surprising that only 1 study has used actigraphy as an outcome measure.^60^ A further advantage of actigraphy is that in addition to standard measures of objective sleep continuity, nonparametric methods of activity analysis can be used as outcome measures; this is important as nonparametric methods of actigraphy are more sensitive to change and may therefore be used as a more sensitive outcome measure in nutritional trials.^105^ If it is appropriate, other technologies could be used to measure sleep, such as ambulatory PSG systems, ear-mounted electrodes, dry-electrode EEG headbands, and noncontact radar technology.^106–108^

## CONCLUSION

Despite the fact that sleep disturbances are a core symptom of menopause, the results of the present systematic review indicate that the majority of the studies that have been conducted are of a low methodological quality and have issues including a lack of preregistration and potentially unsuitable statistical analysis. However, despite the large heterogeneity in the choice of intervention and in study designs, the majority of the identified studies showed that a nutritional intervention improved sleep, and specifically, that the interventions improved subjective sleep. The most promising nutritional interventions include isoflavones, soy or soybean-based interventions, and black cohosh. High-quality, adequately powered, randomized controlled trials of these interventions are now necessary, with appropriate outcome measures. These should be trialed alongside other current or future nutritional interventions or supplements that have a sound mechanistic justification for the improvement of subjective or objective sleep.

## Supplementary Material

nuad113_Supplementary_Data

## Supporting Information

The following Supporting Information is available through the online version of this article at the publisher’s website.

PRISMA 2020 Checklist
